# For whom the bell tolls: psychopathological and neurobiological correlates of a DNA methylation index of time-to-death

**DOI:** 10.1038/s41398-022-02164-w

**Published:** 2022-09-24

**Authors:** Sage E. Hawn, Xiang Zhao, Danielle R. Sullivan, Mark Logue, Dana Fein-Schaffer, William Milberg, Regina McGlinchey, Mark W. Miller, Erika J. Wolf

**Affiliations:** 1 National Center for PTSD at VA Boston Healthcare System, Boston, MA USA; 2 Boston University School of Medicine, Department of Psychiatry, Boston, MA USA; 3 Boston University School of Medicine, Department of Medicine, Biomedical Genetics, Boston, MA USA; 4 Boston University School of Public Health, Department of Biostatistics, Boston, MA USA; 5 Translational Research Center for TBI and Stress Disorders (TRACTS) and Geriatric Research, Educational and Clinical Center (GRECC), VA Boston Healthcare System, Boston, MA USA; 6 Department of Psychiatry, Harvard Medical School, Boston, MA USA; 7 Present Address: Department of Psychology, Old Dominion University, Mills Godwin Bldg (134A), Norfolk, VA USA

**Keywords:** Biomarkers, Genetics, Psychology, Neuroscience

## Abstract

Psychopathology is a risk factor for accelerated biological aging and early mortality. We examined associations between broad underlying dimensions of psychopathology (reflecting internalizing and externalizing psychiatric symptoms), PTSD, and age-adjusted GrimAge (“GrimAge residuals”), a DNA methylation biomarker of mortality risk relative to age. We also examined neurobiological correlates of GrimAge residuals, including neurocognitive functioning, blood-based biomarkers (of inflammation, neuropathology, metabolic disease), and cortical thickness. Data from two independent trauma-exposed military cohorts (*n* = 647 [62.9% male, M_age_ = 52], *n* = 434 [90% male, M_age_ = 32]) were evaluated using linear regression models to test associations between GrimAge residuals, psychopathology, and health correlates. Externalizing psychopathology significantly predicted GrimAge residuals in both cohorts (*p*s < 0.028). PTSD predicted GrimAge residuals in the younger (*p* = 0.001) but not the older cohort. GrimAge residuals were associated with several neurobiological variables available in the younger cohort, including cognitive disinhibition (*p*_adj_ = 0.021), poorer memory recall (*p*_adj_ = 0.023), cardiometabolic pathology (*p*_adj_ < 0.001), oxidative stress (*p*_adj_ = 0.003), astrocyte damage (*p*_adj_ = 0.021), inflammation (C-reactive protein: *p*_adj_ < 0.001; IL-6: *p*_adj_ < 0.001), and immune functioning (*p*_adj_ < 0.001). A subset of inflammatory and neuropathology analytes were available in the older cohort and showed associations with GrimAge residuals (IL-6: *p*_adj_ < 0.001; TNF-α: *p*_adj_ < 0.001). GrimAge residuals were also associated with reduced cortical thickness in right lateral orbitofrontal cortex (*p*_adj_ = 0.018) and left fusiform gyrus (*p*_adj_ = 0.030), which are related to emotion regulation and facial recognition, respectively. Psychopathology may be a common risk factor for elevated mortality risk. GrimAge could help identify those at risk for adverse health outcomes and allow for early disease identification and treatment.

## Introduction

Psychopathology is a risk factor for premature morbidity and mortality [1]. This association has been reported for numerous psychiatric diagnoses, including posttraumatic stress disorder (PTSD) [2], major depressive disorder (MDD) [3], anxiety [4], antisocial personality disorder [5], substance use disorders [6], and alcohol and nicotine dependence [7]. Psychopathology may be a shared risk factor for premature morbidity and mortality, suggesting the potential for common biological pathways linking psychiatric stress to adverse health outcomes.

Shared variance across common psychiatric disorders is reflected in broad, underlying dimensions capturing internalizing and externalizing psychopathology. These underlying spectra of psychopathology offer a useful framework for evaluating shared health burden across individual psychiatric disorders as they may have greater biological relevance than individually defined disorders [8]. Consistent with this, there is evidence that the internalizing dimension, which underlies individual mood and anxiety disorders, is associated with a greater ability to predict mortality risk over a 20-year period relative to individual disorders alone [9]. This suggests that psychiatric distress shared across internalizing disorders is a common factor that increases the risk for premature morbidity and mortality. However, no study has examined if the risk is equivalent for primarily distress-based disorders (e.g., major depression, dysthymia, and generalized anxiety disorder) as compared to fear-based disorders (e.g., panic and phobic disorders), which are the two lower-order factors of the higher-order internalizing dimension. Additionally, although individual externalizing disorders have been shown to predict early death [5], the relative associations between higher-order dimensions of externalizing (e.g., characterized by impulsivity, substance use, and antisociality) in comparison to internalizing have yet to be examined. PTSD shows genotypic [10] and phenotypic [11] associations with both internalizing and externalizing dimensions of psychopathology, highlighting the need to investigate its influence on premature mortality in conjunction with both of these broad dimensions of psychopathology. A better understanding of associations between psychopathology and risk for early death would improve targeted interventions for those at greatest risk. Biomarkers of disease and death are critical tools for assessing risk, tracking change over time, and informing the biological mechanisms that link psychiatric stress to adverse health outcomes. A recently developed blood-based DNA methylation (DNAm) biomarker of mortality risk referred to as “GrimAge” [12] may prove useful for these purposes. GrimAge was developed by first identifying blood-based molecules previously associated with mortality and morbidity, which were (in addition to self-reported smoking pack-years) then regressed on thousands of DNAm loci to generate DNAm-based surrogate biomarkers of these molecules and behaviors. Next, time-to-death due to all-cause mortality was regressed on these surrogate variables, creating a weighted composite score, which was then linearly transformed to be in the unit of years. Regressing the composite DNAm GrimAge score on chronological age yields a residual variable that indexes mortality risk relative to that expected based on age (“GrimAge residuals”). After adjusting for mortality risk factors, GrimAge residuals strongly predicted (*p* = 5.7E-29) lifespan and time to age-related diseases (e.g., heart disease) in initial validation studies in several large, longitudinal epidemiological cohorts (*n* = 6,935) comprising men and women varying of mean age from 56.2 to 67 years [12].

GrimAge differs from prior DNAm-based indicators of biological aging, such as the Horvath [13] and Hannum et al. [14] algorithms, as it was not trained on chronological age, but rather time-to-death. This is important because the DNAm age calculators are so accurate in predicting chronological age (*r*s ~0.90; Wolf et al., 2018) that there is little residual variance left to index accelerated (or decelerated) aging and thus effect sizes for associations between environmental variables and accelerated aging in DNAm tend to be quite small [15]. Though GrimAge and other DNAm age estimates are strongly influenced by chronological age, after partialling out this effect, GrimAge residuals were found to be only weakly correlated (*r*s = 0.00 – −0.02) with Horvath and Hannum residual estimates [16]. An exception to this is PhenoAge [17], another DNAm-based predictor of lifespan, which, when adjusted for chronological age, has been shown to be moderately correlated with age-adjusted GrimAge (*r* = 0.40) [16]. Still, GrimAge outperforms each of these other DNAm-based biomarkers in its prediction of time-to-death and other health-related metrics [12, 16].

In addition to its ability to predict lifespan and healthspan, age-adjusted GrimAge has been associated with neurodegeneration and other age-related changes in brain health. In a recent study of 709 elderly Scottish individuals [18], higher GrimAge residuals (indexing greater risk of death than would be expected based on age) were associated with poorer neurocognitive functioning and decreased neural integrity, including decreased brain volume and increased volume of vascular brain lesions. Further, a recent study of trauma-exposed individuals reported that higher GrimAge residuals were associated with reduced cortical thickness in the right lateral orbitofrontal cortex and the right posterior cingulate cortex [19]. Follow-up analyses indicated the right lateral orbitofrontal cortex effect was evident among individuals with PTSD, but not controls. Thus, trauma exposure and psychopathology may increase the risk for epigenetic aging and associated neuropathology.

Consistent with this, higher GrimAge residuals have been reported among PTSD [19, 20] and MDD [21] cases compared to controls. PTSD symptom course has also been associated with changes in GrimAge residuals over time [20]. Effect sizes for psychiatric cases vs. controls have been markedly greater in studies examining GrimAge residuals (e.g., Cohen’s *d*s = 0.54–0.64) [20, 21] compared to studies focused on other DNAm age residuals (e.g., Cohen’s *d*s = 0.18–0.42) [22, 23].

It is important to comprehensively examine the clinical utility of GrimAge by evaluating its associations with a range of age-related neurobiological factors, such as neural integrity, cognitive functioning (e.g., inhibitory control, verbal memory recall), and biomarkers of neuropathology, inflammation, oxidative stress, immune, and metabolic functioning, all of which are associated with aging and mortality [15, 24–26]. Recent advancements in Single Molecule Array (Simoa®) technology have resulted in the ability to digitally measure analytes at very low concentrations (e.g., sub-picomolar lower limits of detection) with a high degree of precision, maximizing early detection of biomarkers associated with health outcomes that occur many years later. For example, several Simoa® neuropathology biomarkers (tau, neurofilament light chain, amyloid-ß species) have demonstrated the ability to predict later conversion to Alzheimer’s disease among individuals without current symptoms [27]. GrimAge has yet to be examined in association with these high sensitivity early markers of inflammation and neuropathology. Testing these associations is essential for risk prediction and reduction, particularly among young individuals for whom the capacity to alter disease course via prevention and intervention efforts may be most successful.

The primary aim of this study was to examine associations between internalizing and externalizing psychopathology dimensions and PTSD with GrimAge residuals in two independent cohorts. We hypothesized that GrimAge residuals would evidence positive associations with PTSD and the internalizing and externalizing psychopathology dimensions. The second aim was to examine the neurobiological correlates of shortened time-to-death, as indexed by GrimAge residuals. We expected GrimAge residuals to be associated with: (a) greater pathology in neurologic (neurofilament light, glial fibrillary acidic protein [GFAP], tau, brain-derived neurotrophic factor [BDNF], amyloid ß 40 and 42, phosphorylated neurofilament heavy chain, neuron-specific enolase [NSE]), inflammatory (C-reactive protein [CRP], interleukin 6 and 10 [IL-6, IL10], eotaxin, tumor necrosis factor [TNF-α]), immune (white blood cells), metabolic (e.g., blood pressure, triglycerides, insulin), and oxidative stress (gamma-glutamyl transferase [GGT]) markers; (b) neurocognitive deficits; and (c) reduced cortical thickness in frontal regions.

## Materials and methods

### Participants

#### National Center for PTSD (NCPTSD) cohort

The first cohort was comprised of 647 participants with DNAm data enrolled in one of two VA NCPTSD studies with identical comprehensive psychiatric diagnostic assessments, allowing the data to be combined. The NCPTSD cohort was comprised of trauma-exposed veterans and a subset of their spouses; the cohort was majority male (62.9%) with a mean age of 52 years (SD = 10.6). A detailed description of these protocols is provided in Logue et al. [28].

#### Translational Research Center for TBI and stress disorders (TRACTS) cohort

The second cohort (*n* = 434) consisted of young (*M*_age_ = 32.4, SD = 8.6) primarily male (90%) US veterans enrolled in a large, ongoing prospective study of psychological and brain trauma associated with the post-9/11 conflicts (The Translational Research Center for TBI and Stress Disorders/TRACTS) [29]. Participants completed a comprehensive psychiatric evaluation, including neurocognitive and psychological assessments. Participants also provided blood samples for genetic and metabolic testing and underwent magnetic resonance imaging (MRI) of the brain. Individuals with moderate-to-severe traumatic brain injury (TBI; *n* = 15) were excluded from analyses investigating neurobiological correlates of GrimAge. The VA Boston Institutional Review Board approved all study procedures in both cohorts.

### Measures

#### Psychiatric measures (Both cohorts)

In both cohorts, PTSD was assessed with the Clinician-Administered PTSD Scale (CAPS) [30], a structured diagnostic interview assessing the frequency and intensity of *DSM-IV* PTSD symptom criteria. Frequency and intensity ratings were summed to create a dimensional score of lifetime PTSD severity. In both cohorts, lifetime Axis I disorders were assessed using the Structured Clinical Interview for *DSM-IV* (SCID-IV) [31]. In the NCPTSD cohort, all items were administered without applying standard “skip-out” rules, permitting both symptom count scores and diagnostic determinations. In the TRACTS cohort, only diagnostic determinations were available for analysis as administration of the SCID followed the standard skip-out procedures. Antisocial personality disorder (ASPD) was assessed in the NCPTSD cohort only using the SCID for Personality Disorders [32] and the International Personality Disorders Exam [33], which were harmonized following the procedure detailed in Miller et al. [34] (see also Supplementary Materials).

#### GrimAge (Both cohorts)

DNA was isolated from peripheral blood samples. In both cohorts, genotypes were assayed on the Illumina HumanOmni2.5–8 array and DNAm on the Illumina Infinium MethylationEPIC array. Genotyping and DNAm quality control methods are described in the Supplementary Materials, as are the calculation procedures for GrimAge, other DNAm age estimates, and ancestry principal components (PCs). An index of age-adjusted GrimAge was created by regressing GrimAge on chronological age and saving the residuals (“GrimAge residuals”). Conceptually, positive residual values index mortality risk that is elevated compared to chronological age, while negative residual values suggest reduced mortality risk relative to age. Blood sample white blood cell proportions (CD8-T and CD4-T cells, natural killer cells, b-cells, monocytes) were calculated from the methylation data [35, 36] and included as covariates (technical confounders) in analyses with GrimAge residuals as the outcome. As cell type composition may be confounded with DNAm, even when DNAm-based variables are the exogenous variable, we created a second GrimAge residual variable for use in follow-up analyses of models in which GrimAge was the independent variable in which variance associated with chronological age and cell type estimates was regressed out from raw GrimAge estimates.

#### Neuropsychological measures (TRACTS cohort)

We examined age-related neurocognitive performance in two domains: inhibitory control and verbal memory. Two measures of inhibitory control were analyzed: total commission errors on the Affective Go/No-go task (AGNG) [37] and inhibition scores on the color-word interference test (i.e., Stroop) from the Delis–Kaplan Executive Function System [38]. Verbal memory recall was assessed using the California Verbal Learning Test: Second Edition (CVLT-II) [39], in which participants’ recall of a 16-item word list across five learning trials was summed. Additional information is provided in the Supplementary Materials.

#### Metabolic and associated analytes acquired through clinical lab assays (TRACTS cohort)

We used standard measures of metabolic pathology (e.g., height, weight, cholesterol, and blood pressure) to calculate a latent metabolic pathology variable and associated factor scores (Supplementary Materials and Table S1). Higher scores index greater metabolic pathology and metabolic syndrome (MetS) [40]. Total measured (as opposed to estimated from DNAm) white blood cells (WBCs) and log-transformed C-reactive protein (CRP) values were used to assess immune response and inflammation, respectively, and log-transformed gamma-glutamyl transferase (GGT) was evaluated as an index of systemic oxidative stress. These blood samples were processed and shipped the same day of the blood draw to a clinical laboratory (Quest Diagnostics, Cambridge, MA). Standardization procedures followed those set by the College of American Pathologists.

#### Simoa-derived neurologic and inflammatory analytes (both cohorts)

Neurology markers (neurofilament light, GFAP, tau, BDNF, amyloid ß 40 and 42, phosphorylated neurofilament heavy chain, and NSE) and inflammation analytes (IL-6, IL10, eotaxin, and TNF-α) were derived from plasma (from the same blood draw as the other blood-based biomarkers) using Simoa® bead technology in the TRACTS cohort (Quanterix, Billerica, MA). A subset of these markers (IL-6, IL10, TNF-α, NSE, and BDNF) were also available in the NCPTSD cohort. Additional information regarding cohort-specific Simoa® methodology is available in the Supplementary Materials. Simoa® markers were log-transformed and outliers >3 SD ± the mean removed.

#### MRI data acquisition and processing (TRACTS cohort)

Structural imaging data were available for 389 participants in the TRACTS cohort. Two Magnetization Prepared Rapid Gradient Echo (MP-RAGE) T1-weighted structural scans were acquired on a 3-Tesla Siemens Trio whole-body TIM Trio MRI scanner (*n* = 361) or a Siemens Prisma scanner with Syngo D13D software (*n* = 28). The two T1-weighted structural scans were averaged to create a single high contrast-to-noise image. A scanner flag was included in analyses as a covariate to account for potential scanner differences. Cortical thickness analysis was performed using the FreeSurfer image analysis suite (version 5.3, http://surfer.nmr.mgh.harvard.edu; see Supplementary Materials).

### Data analysis

In both cohorts, associations between GrimAge residuals and residuals from other estimates of DNAm age (Horvath, Hannum, and Levine/DNAmPheno Age) were first evaluated using correlational analyses. In the NCPTSD cohort, psychiatric symptom scores were summed to create dimensional scores for each diagnosis assessed via the SCID, and included in a confirmatory factor analysis to model the fear, distress, and externalizing latent variables. Because PTSD shows associations with both internalizing and externalizing dimensions of psychopathology and has previously been associated with advanced epigenetic age [15], it was not included in the confirmatory factor analysis, but was instead included in regression models alongside the latent psychopathology factor scores. Indicators for each latent variable are notated in Table S2 and fit statistics are provided in Table S1 (see also [35]). We then evaluated these three psychopathology factor scores and PTSD severity as predictors of GrimAge residuals in a hierarchical linear regression model, controlling for the top three global ancestry PCs, sex, and estimated WBCs (from the DNAm data) in the first step of the regression. Next, we followed up on significant factor score effects by evaluating the individual diagnoses that comprised the factor. We examined if effects observed in the NCPTSD cohort replicated in the TRACTS cohort. As the TRACTS cohort did not assess all diagnoses included in the NCPTSD cohort (e.g., ASPD), we were limited to evaluating associations between GrimAge residuals and the available individual diagnoses. Covariates replicated those included in the NCPTSD analyses.

Examination of health and biological correlates of GrimAge residuals proceeded in each cohort based on the available data, such that neuropsychological, metabolic, MRI, and Simoa® markers were examined in TRACTS and a subset of Simoa® markers were examined in NCPTSD. We evaluated if GrimAge residuals were associated with neurocognitive deficits that are commonly observed in aging—disinhibition and verbal memory recall—in three regressions with age and sex included as covariates. A False discovery rate (FDR) adjusted *p* value (*p*_adj_, or *q* value) was used to account for multiple testing across the three neuropsychological outcomes.

We next examined GrimAge residuals in association with the blood-based biomarkers. Linear regression analyses controlling for age and sex were used to test whether GrimAge residuals predicted the following separately analyzed outcomes: MetS factor scores, CRP, GGT, total measured (not estimated) WBC, four Simoa® markers associated with inflammation (IL-6, IL10, eotaxin, and TNF-α), and eight Simoa® markers associated with neurodegeneration (neurofilament light, GFAP, tau, BDNF, amyloid ß 40 and 42, phosphorylated neurofilament heavy chain, and NSE). A smaller subset of Simoa® markers (IL-6, IL10, TNF-α, BDNF, and NSE) were available in the NCPTSD cohort for replication analyses. FDR was used to correct for multiple testing across 16 blood biomarker outcomes in TRACTS and five in NCPTSD.

We evaluated if previously identified associations between GrimAge residuals and cortical thickness in regions associated with emotion- and threat-regulation [19] replicated in our sample. To do so, measures of right and left hemisphere orbitofrontal and posterior cingulate cortical thickness were submitted to an omnibus hierarchical linear regression analysis in which sex, age, and scanner were entered in an initial model and GrimAge residuals were entered in the second step of the model. Multiple testing adjusted *p* values for GrimAge residual main effect analyses were determined using Monte Carlo null simulation with 10,000 replicates in which GrimAge residuals were randomly permuted between subjects [41]. The analysis imposed multiple-testing control while taking into account the correlations between the cortical thickness scores.

Finally, the main effect of the GrimAge residuals was also investigated in a follow-up unbiased whole brain cortical thickness analysis (*n* = 361) in the TRACTS cohort. The Freesurfer version 7.1 command line tools mris_preproc, mri_surf2surf, and mri_glmfit and spatial smoothing of 10 mm full-width half-maximum were used. Age, sex, and scanner were included in the model as covariates. The command line tool mri_glmfit-sim was used to correct for multiple comparisons with a vertex-wise/cluster forming threshold of *p* < 0.0001 and a cluster-wise *p* value of *p* < 0.05. Significant regions were extracted for analyses featuring additional covariates.

Sensitivity analyses featuring additional covariates and GrimAge residuals adjusted for both age and cell type composition were performed on FDR significant outcomes and are reported in the Supplementary Materials.

## Results

### Descriptive statistics

Participant characteristics and descriptive statistics for the NCPTSD and TRACTS cohorts are listed in Table 1.

**Table 1:** Participant characteristics and descriptive statistics.

	NCPTSD (*N* = 647)		TRACTS (*N* = 434)	
Variable	*M* (SD)	*n* (%)	*M* (SD)	*n* (%)
Demographics				
Sex (male)		407 (62.9)		392 (90.3)
Age	51.85 (10.60)		32.44 (8.63)	
Race				
White		525 (81.1)		322 (74.2)
Black/African American		80 (12.4)		37 (8.5)
Asian		8 (1.2)		6 (1.4)
American Indian/Alaska Native		57 (8.8)		3 (0.7)
Hawaiian/Other Pacific Islander		2 (0.3)		2 (0.5)
Unknown		38 (5.9)		2 (0.5)
Psychiatric				
Lifetime PTSD severity/Dx	60.12 (32.93)	389 (60.1)	69.00 (32.98)	351 (76.8)
# of Trauma types	9.90 (4.26)		1.95 (1.80)	
Lifetime AUD severity/Dx	8.03 (7.81)	376 (58.8)		286 (65.9)
Lifetime Non-AUD SUD severity/Dx	5.24 (9.53)	191 (29.7)		137 (31.6)
Lifetime ASPD severity/Dx	5.19 (5.80)	22 (4.8)		
Neuropsychological				
AGNG				11.56 (9.19)
Stroop^b^				26.59 (12.14)
CVLT-II^c^				48.33 (9.88)
Biological				
CRP (log)			−0.77 (0.29)	
GGT (log)			1.39 (0.24)	
WBC			6.28 (1.71)	
AB40 (log)			2.32 (0.07)	
AB42 (log)			0.91 (0.08)	
BDNF (log)	3.41 (0.38)		3.10 (0.47)	
GFAP (log)			1.79 (0.16)	
NFL (log)			0.72 (0.19)	
NSE (log)	4.32 (0.26)		4.07 (0.21)	
TAU4 (log)			0.16 (0.23)	
PNF (log)			1.41 (0.35)	
Eotaxin (log)			1.61 (0.14)	
IL10 (log)	−0.10 (0.26)		−0.16 (0.21)	
IL6 (log)	0.19 (0.31)		0.14 (0.27)	
TNF-α (log)	0.53 (0.20)		0.43 (0.13)	

*NCPTSD* National Center for PTSD cohort, *TRACTS* Translational Research Center for TBI and stress disorders cohort, *M* mean, *SD* standard deviation, *PTSD* posttraumatic stress disorder, *dx* diagnosis, *CRP* C-reactive protein, *GGT* gamma-glutamyl transferase, *WBC* total measured white blood cell counts, *AGNG* affective go/no-go task, *CVLT-II* California verbal learning test second edition, *AB40/AB42* amyloid ß 40/42, *BDNF* brain-derived neurotrophic factor, *GFAP* glial fibrillary acidic protein, *NFL* neurofilament light, *NSE* neuron-specific enolase, *PNF* phosphorylated neurofilament heavy, *IL10/6* interleukin 6/10, *TNF-α* tumor necrosis factor.

^a^Raw scores for total commission error = Total positive commissions + total negative commissions.

^b^Scaled scores adjusted for performance = Inhibition – (Color naming + Word reading)/2.

^c^Total words recalled across five learning trials.

### GrimAge, chronological age, and other DNAm age estimates in both cohorts

In the NCPTSD cohort, GrimAge estimates were highly correlated with age (*r* = 0.88, *p* < 0.001) and with Horvath, Hannum, and PhenoAge DNAm age (*r* range = 0.78–0.83, all *p*s < 0.001). Despite high correlations among the raw DNAm measures, GrimAge residuals showed weak associations with Horvath, Hannum, and PhenoAge DNAm age residuals (*r* range = 0.14–0.24, *p* < 0.001), meaning that they shared a maximum 5.67% of the variance with GrimAge residuals. The overall pattern of results was similar in the TRACTS cohort, except that GrimAge residuals were not significantly correlated with Horvath residuals (see Table S3).

### NCPTSD cohort: GrimAge residuals, PTSD, and psychopathology

In the multiple regression model, externalizing factor scores were significantly associated with GrimAge residuals (b = 0.35, standardized [std] β = 0.35, *p* < 0.001). PTSD severity and the fear and distress factor scores were not associated with GrimAge residuals (Table 2). Follow-up analyses examining each component of the externalizing spectrum revealed that non-alcohol use substance use disorders (SUD; b = 0.11, std β = 0.20, *p* < 0.001) and ASPD (b = 0.10, std β = 0.13, *p* = 0.017) predicted GrimAge residuals, but lifetime alcohol use disorders (AUD) did not (b = 0.05, std β = 0.08, *p* = 0.107). These effects remained significant in sensitivity analyses (see Supplementary Materials).

**Table 2:** Psychopathology as a predictor of GrimAge residuals in both cohorts.

*NCPTSD*				
Variable	β	B	*SE*	*p*
Covariates:				
PC1	0.075	9.148	4.616	**0.048**
PC2	−0.023	−2.722	4.443	0.540
PC3	−0.003	−0.397	4.594	0.931
CD8-T	0.064	7.720	4.646	0.097
CD4-T	−0.038	−3.163	3.245	0.330
NK	−0.241	−23.402	3.566	**<0.001**
B-cell	−0.145	−16.815	4.304	**<0.001**
Monocytes	−0.044	−7.601	6.932	0.273
Sex	0.344	3.178	0.359	**<0.001**
Primary Predictors				
Externalizing Factor Score	0.345	0.351	0.044	**<0.001**
Distress Factor Score	−0.120	−0.178	0.095	0.062
Fear Factor Score	0.018	0.008	0.025	0.738
Lifetime PTSD Severity	0.056	0.008	0.006	0.215
***TRACTS***				
Covariates:				
PC1	−0.186	−15.297	3.984	**<0.001**
PC2	0.022	2.064	4.485	**<0.001**
PC3	0.053	4.814	4.288	0.262
CD8-T	−0.233	−20.689	4.174	**<0.001**
CD4-T	−0.144	−9.883	3.346	**0.003**
NK	−0.238	−23.583	4.639	**<0.001**
B-cell	−0.051	−7.153	7.010	0.308
Monocytes	−0.108	−14.293	6.473	**0.028**
Sex	−0.150	−1.691	0.527	**0.001**
Primary Predictors:				
Lifetime AUD dx	0.077	0.541	0.339	0.112
Lifetime non-AUD SUD dx	0.104	0.747	0.339	**0.028**
Lifetime PTSD dx	0.149	1.165	1.220	**0.001**

Results based on hierarchical regression models with covariates entered into the first step and psychopathology variables entered in the second. The △*R*^2^ coefficients were significant for the second step across models.

*PC* principal component, *CD* cluster of differentiation, *NK* natural killer, *PTSD* posttraumatic stress disorder, *AUD* alcohol use disorder, *SUD* substance use disorder, *dx* diagnosis, *β* standardized beta, *B* unstandardized beta, *SE* standard error for unstandardized beta.

Bolded values reflect *p* < 0.05.

### TRACTS cohort: GrimAge residuals, externalizing disorders, and PTSD

Consistent with findings from the NCPTSD cohort, lifetime non-alcohol-use SUD significantly predicted GrimAge residuals (b = 0.75, std β = 0.10, *p* = 0.028), but lifetime AUD did not (b = 0.541, std β = 0.08, *p* = 0.112) (Table 2). Lifetime PTSD diagnosis was associated with GrimAge residuals (b = 1.17, std β = 0.15, *p* = 0.001). These effects remained significant in sensitivity analyses (see Supplementary Materials).

### TRACTS cohort: GrimAge residuals and neuropsychological constructs

GrimAge residuals were associated with greater errors on the AGNG task (b = 0.38, std β = 0.14, *p*_adj_ = 0.021; Table 3). This effect remained significant in follow-up analyses (see Supplementary Materials). GrimAge residuals were also associated with poorer verbal memory recall on the CVLT (b = −0.372, std β = −0.12, *p*_adj_ = 0.023; Table 3). This effect missed the threshold for significance (*p* = 0.065) in follow-up analyses (see Supplementary Materials). GrimAge residuals and Stroop scores were not significantly associated (b = 0.35, std β = 0.05, *p*_adj_ = 0.318).

**Table 3:** GrimAge residuals as a predictor of neuropsychological constructs.

	AGNG			Stroop			CVLT-II		
Variable	β	*p*	*p*_*adj*_	β	*p*	*p*_*adj*_	β	*p*	*p*_*adj*_
Covariates:									
Sex	0.011	0.835	–	−0.003	0.955	–	0.033	0.518	–
Age	0.006	0.901	–	−0.041	0.429	–	−0.171	**0.001**	–
Primary Predictor:									
GrimAge Residuals	0.136	0.007	**0.021**	0.052	0.318	0.318	−0.122	0.015	**0.023**

Results based on hierarchical regression models with covariates entered into the first step and GrimAge Residuals entered in the second. △*R*^2^ coefficients were significant for the second step across models with significant second step effects.

*AGNG* affective go/no-go task, *CVLT-II* California verbal learning test: second edition, *PTSD* posttraumatic stress disorder, *AUD* alcohol use disorder, *MDD* major depressive disorder; *p*_adj_ *p* value adjusted for multiple testing by controlling the false discovery rate (FDR) of 5%.

Bolded values reflect *p* or *p*_adj_ < 0.05.

### TRACTS cohort: GrimAge residuals and metabolic, immune, and neurology analytes

As shown in Table 4, GrimAge residuals were significantly associated with the MetS factor scores (b = 0.002, β = 0.139, *p*_adj_ < 0.001), CRP (b = 0.020, β = 0.220, *p*_adj_ < 0.001), GGT (b = 0.012, β = 0.161, *p*_adj_ = 0.003), and total measured WBCs (b = 0.167, β = 0.324, *p*_adj_ < 0.001). With regard to Simoa® markers, GrimAge residuals were significantly associated with GFAP (b = −0.007, β = −0.133, *p*_adj_ = 0.021) and two inflammatory markers: Eotaxin (b = 0.005, β = 0.111, *p*_adj_ = 0.050) and IL-6 (b = 0.023, β = 0.286, *p*_adj_ < 0.001). All effects remained significant in follow-up analyses (see Supplementary Materials).

**Table 4:** Associations between GrimAge residuals and metabolic, immune, and neurology markers.

	TRACTS			NCPTSD		
Variable	β	*p*	*p*_*adj*_	β	*p*	*p*_*adj*_
**Metabolic, Immune, and Oxidative Stress**						
MetS	0.139	<0.001	**<0.001**	–	–	–
WBC	0.324	<0.001	**<0.001**	–	–	–
GGT	0.161	0.001	**0.003**	–	–	–
**Inflammatory Markers**						
CRP	0.220	<0.001	**<0.001**	–	–	–
Eotaxin	0.108	0.026	0.059	–	–	–
IL10	0.072	0.165	0.264	0.049	0.281	0.281
IL6	0.286	<0.001	**<0.001**	0.341	<0.001	**<0.001**
TNF-alpha	0.077	0.135	0.240	0.156	<0.001	**<0.001**
**Neurologic Markers**						
GFAP	−0.132	0.008	**0.021**	–	–	–
NFL	−0.055	0.266	0.387	–	–	–
pNF	0.001	0.985	0.985	–	–	–
AB42	0.052	0.316	0.421	–	–	–
AB40	0.080	0.122	0.240	–	–	–
NSE	0.043	0.391	0.447	0.072	0.107	0.143
BDNF	0.043	0.386	0.447	0.071	0.114	0.143
Tau4	0.025	0.661	0.705	–	–	–

Results based on hierarchical regression models controlling for age and sex (covariate effects not shown for simplicity). The △*R*^2^ coefficients were significant for the second step across models with significant second step effects.

*MetS* metabolic syndrome, *WBC* total measured white blood cell count, *GGT* gamma-glutamyl transferase, *CRP* C-reactive protein, *IL10/IL6* interleukin 10/6, *TNF-alpha* tumor necrosis factor, *GFAP* glial fibrillary acidic protein, *NFL* neurofilament light, *pNF* phosphorylated neurofilament heavy chain, *AB42/AB40* amyloid beta 40/42, *NSE* neuron-specific enolase, BDNF brain-derived neurotrophic factor, *p*_adj_ *p* value adjusted for multiple testing by controlling the false discovery rate (FDR) of 5%.

Bolded values reflect *p*_adj_ < 0.05.

### NCPTSD Cohort: GrimAge residuals and immune and neurology analytes

As shown in Table 4, the effect of GrimAge residuals on IL-6 that was observed in the TRACTS cohort was replicated in the NCPTSD cohort (b = 0.022, β = 0.317, *p*_adj_ < 0.001). GrimAge residuals were also associated with TNF-α levels in the NCPTSD cohort (b = 0.007, β = 0.156, *p*_adj_ < 0.001). All effects remained significant in follow-up analyses (see Supplementary Materials).

### TRACTS cohort: GrimAge residuals and neural integrity

The analysis evaluating associations between GrimAge residuals and cortical thickness in two frontal regions of interest revealed that GrimAge residuals were negatively associated with cortical thickness in the right lateral orbitofrontal cortex (b = −0.006, *p*_adj_ = 0.018; Table 5). This effect remained significant in the sensitivity analyses (see Supplementary Materials).

**Table 5:** Associations between GrimAge residuals and cortical thickness in regions of interest.

	RH OFC			LH OFC			RH PCC			LH PCC		
Variable	b	*p*	*p*_adj_	b	*p*	*p*_adj_	b	*p*	*p*_adj_	b	*p*	*p*_adj_
Covariates:												
Scanner	0.051	0.065	--	0.033	0.220	--	−0.020	0.455	--	0.011	0.699	--
Sex	−0.022	0.401	--	−0.039	0.140	--	0.021	0.428	--	−0.018	0.513	--
Age	−0.007	**0.000**	--	−0.007	**0.000**	--	−0.006	**0.000**	--	−0.007	**0.000**	--
Primary predictor:												
GrimAge residuals	**−**0.006	0.007	**0.016**	−0.005	0.035	0.116	0.003	0.184	0.498	0.000	0.850	1.00

Results based on hierarchical regression models with covariates entered into the first step and GrimAge residuals entered in the second. The △*R*^2^ was significant for the second step of the model predicting RH OFC.

*RH* right hemisphere, *LH* left hemisphere, *OFC* orbitofrontal cortex, *PCC* posterior cingulate cortex, *p*_adj_ *p* value adjusted for multiple testing.

Bolded values reflect *p* or *p*_adj_ < 0.05.

**Fig. 1:**
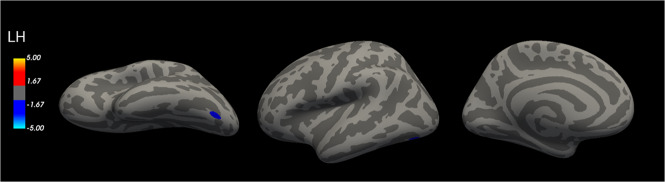
Significant whole brain cortical thickness results. In a whole brain cortical thickness analysis with a vertex-wise threshold of *p* < 0.0001 and cluster corrected at *p* < 0.05, results revealed that there was a significant negative association between GrimAge residuals and the left fusiform gyrus. The color bar indicates the log 10 value for *p* values associated with the cluster-corrected results. LH = left hemisphere.

We also examined whole brain cortical thickness associations with GrimAge residuals. There was a significant negative association between GrimAge residuals and whole brain cortical thickness in the left fusiform gyrus that survived multiple comparison correction (peak MNI coordinates = −40.6 −73.0 −15.1, peak value = −4.876, number of vertices = 136, cluster size = 91.88 mm^2^, voxel-wise *p* < 0.0001, cluster-corrected *p* < 0.03; Fig. 1). This remained significant in the sensitivity analyses (see Supplementary Materials).

## Discussion

Elucidating the association between psychopathology and risk for early death is crucial for intervention and prevention efforts. Reliable biomarkers of disease and mortality risk offer promising insight into this association and have the potential to serve as useful clinical tools for identifying underlying mechanisms and assessing risk and intervention efforts. We examined associations between the most reliable biomarker of age-independent time-to-death to date, GrimAge, broad dimensions of psychopathology, and PTSD, and evaluated the neurobiological correlates of GrimAge. We found that GrimAge residualized for chronological age was associated with externalizing psychopathology, PTSD, and a wide range of age-related neurocognitive and neurobiological biomarkers, which suggests the clinical value of this DNAm index.

### Psychopathology and shortened time-to-death

This is the first study to investigate GrimAge in association with broad dimensions of psychopathology. Results demonstrated associations between externalizing psychopathology and GrimAge residuals. These associations extended to two of the individual components (i.e., non-alcohol SUD, ASPD) of the broader externalizing dimension in the NCPTSD cohort and the effect for non-alcohol SUD replicated in the independent TRACTS cohort of young veterans. Notably, the magnitude of the effect for the externalizing dimension (std β = 0.35) was roughly double that for the individual disorders comprising this spectrum, suggesting the biological relevance and statistical advantages of modeling this common factor.

These results are consistent with prior research suggesting that individual externalizing disorders are associated with elevated risk for premature all-cause mortality [5]. We also found a positive association between PTSD and GrimAge residuals in the TRACTS cohort (though not the NCPTSD cohort), replicating prior work for this disorder [19, 20]. Together, this highlights the critical need for assessment of externalizing characteristics among trauma-exposed samples. Whereas focus has historically been placed on the risk of death related to behavior-based externalizing features (e.g., accidental, drug-related, death by suicide [42]), our findings suggest a possible differential *biological* course to shortened time-to-death for externalizing psychopathology relative to internalizing. This, in conjunction with the greater effect size compared to what was shown for individual disorders, is consistent with research suggesting that broad dimensions of psychopathology that reflect common features of individual disorders may offer an enhanced ability to identify biomarkers and biological consequences of such psychopathology [8]. This was further supported by our findings that GrimAge residuals were associated with a wide variety of biomarkers spanning inflammation, oxidative stress, metabolic dysregulation, and neuropathology.

### GrimAge residuals and neurocognitive function

The link between shortened time-to death and externalizing psychopathology in this study was consistent with our results demonstrating an association between GrimAge residuals and disinhibition, as disinhibition is at the core of externalizing disorders [43]. Given the cross-sectional nature of the data, the directionality between these variables remains unclear. It is possible that disinhibition increases the risk for early mortality, and/or that GrimAge is a biomarker for reduced cognitive functioning in this domain. Consistent with prior work demonstrating associations between GrimAge residuals and overall cognitive decline [18], we found that GrimAge residuals were sensitive to worse verbal memory, suggesting GrimAge could be useful in identifying individuals who would benefit from early cognitive interventions to preserve functioning.

### GrimAge residuals and blood-based biomarkers

GrimAge residuals showed associations with a broad range of blood-based biomarkers implicated in age-related disease and premature mortality risk, including inflammation (CRP, WBC, IL-6, and TNF-α), oxidative stress (GGT), neuropathology (GFAP), and metabolic disease (MetS). One particularly novel finding is the association between GrimAge residuals and GGT, a marker of oxidative stress. Oxidative stress, a molecular mechanism fundamental to aging that is frequently implicated in disease processes, leads to the depletion of antioxidants when chronically elevated, eventually resulting in cell degeneration and apoptosis [44]. GGT is a biomarker of cardiometabolic disease, MetS, and mortality risk [45], and elevated GGT is an index of liver disease and alcohol abuse [46]. Thus, GGT could be a mechanism underlying the pathway between externalizing psychopathology and early death.

This is the first study to demonstrate associations between GrimAge residuals and ultra-sensitive Simoa® markers of inflammation and neurodegeneration. Prior research has failed to find associations between GrimAge residuals and proinflammatory cytokines, but these were assayed using typical ELISA-based methods that are less sensitive to low levels of the analyte [20]. The effect of GrimAge residuals on IL-6 was replicated across our two independent cohorts. These results highlight the value of Simoa® in detecting what may be very early risk markers for disease, even in a relatively young adult cohort. GrimAge was sensitive to these inflammatory biomarkers and has the potential to be used as a tool for identifying preclinical individuals and preventing premature mortality.

To that end, GrimAge residuals were associated with GFAP, a marker of astrocytic damage [47]. As GFAP is expressed primarily in the brain, only trace or undetectable levels of GFAP are usually observed in the blood. When GFAP is found in blood, it is typically considered an index of astrocytic damage and disruption of blood–brain barrier integrity [48]. This can occur in response to brain damage, degeneration of the central nervous system (CNS), or aging [47]. In this study, GrimAge residuals were negatively associated with GFAP levels, which is the opposite direction of effect as was hypothesized. That said, prior work has reported low to very low levels of blood-based GFAP in patients with a variety of neurological diseases, despite the presence of neurological symptoms [48]. It is possible that rapid astroglial destruction (as in the acute aftermath of severe TBI or an intracerebral hemorrhage) may be required to observe increases in GFAP in blood [48]. We would not expect to see evidence of this in our cohort, as veterans with moderate-to-severe TBI were excluded from these analyses. Although GFAP upregulation is linked to a range of acute and chronic neuropathologies, there may be neuroprotective effects associated with increased GFAP [49, 50], as its function is highly context-and development specific; under certain circumstances, increased GFAP can elicit neurogenesis [50]. Additional evaluation of the association between GFAP and GrimAge is necessary to better understand the potential role of GFAP in shortened time-to-death and the implications for altering the course of neurodegenerative diseases.

### GrimAge residuals and cortical thickness

GrimAge residuals were associated with reduced thickness in the right lateral orbitofrontal cortex, replicating prior results by Katrinli et al. [19] and consistent with the evidence of frontal cortical thinning with age [51]. The lateral orbitofrontal cortex is important for inhibitory control [52], including regulation of emotion and threat detection [53], and it could be that reduced cortical thickness in this region partially contributes to the behavioral disinhibition commonly observed in externalizing psychopathology.

A follow-up whole brain cortical thickness analysis further suggested that GrimAge residuals were associated with reduced cortical thickness in the left fusiform gyrus (FFG). The FFG is well known for its role in facial, object, and word recognition [54]. Evidence also suggests it is implicated in dementia and aging. Specifically, left FFG atrophy is evident among patients with semantic dementia [55], with one study identifying the left FGG as the hub of the neuroanatomical network predicting semantic processing among patients with semantic dementia [56]. FFG volume has also been shown to decrease over time as part of the healthy aging process [57]. The association between GrimAge residuals and the integrity of this region in this study further suggests the clinical relevance of the GrimAge algorithm and indicates that monitoring semantic functioning, starting in young adulthood, among those with advanced GrimAge may be warranted.

### Limitations

Our findings should be considered in the context of several limitations. First, our study was based on cross-sectional data, precluding causal interpretation of the direction of effects. Second, cohorts were primarily male, white, veteran participants, limiting generalizability. That said, associations between GrimAge residuals and externalizing disorders were demonstrated in two independent cohorts of varying mean age, highlighting the utility of GrimAge as a marker of increased risk for early death, even among young individuals. Third, except for a subset of Simoa® markers that were available in both cohorts, we were unable to test for replication of many of the health correlates as most of these data were only collected in the TRACTS cohort.

## Conclusions

These results contribute to burgeoning literature suggesting a meaningful role of GrimAge in the prediction of premature mortality and morbidity. To date, GrimAge is unmatched in its ability to predict age-related disease and time-to-death. Our findings highlight the critical need for assessment of externalizing characteristics among trauma-exposed samples. The consistency of associations between GrimAge residuals and related constructs (i.e., externalizing psychopathology, behavioral disinhibition, frontal cortical thinning) and a range of other neurobiological markers further support the clinical relevance of this DNAm-based index. Given behavioral inhibition and externalizing pathology tend to decrease with age [58, 59], these associations may hold particular relevance for younger veterans. Notably, by using novel biomarker technology that results in a 1000-fold increase in immunoassay sensitivity, this study is the first to detect some of the earliest neurobiological correlates of elevated mortality risk per DNAm patterns. This work has important clinical implications, as lifestyle interventions have been shown to reverse other DNAm-based metrics of biological aging [60] and might also mitigate the risk of premature death. Replication among longitudinal and age-varying cohorts is needed to address questions of generalizability and directionality. Doing so would also inform whether interventions that reduce externalizing behaviors alter neurobiology and mortality risk over a given period of time. Additionally, longitudinal mediation analyses could shed light on whether the various biomarkers significantly associated with GrimAge residuals in these data (e.g., GGT) serve as mechanisms along the pathway between externalizing psychopathology and early death. In conclusion, GrimAge may be a useful tool in identifying those at greatest risk for early mortality, thereby providing opportunities for disease treatment and, with early identification, disease prevention.

## Supplementary information

Supplementary Materials

## Data Availability

Qualified investigators can apply to the PTSD Genetics and TRACTS data repositories to gain access to these data via a Data Use Agreement. Please contact Drs. Miller and McGlinchey regarding access to PTSD Genetics (NCPTSD) and TRACTS data repositories, respectively.

## References

[1] Felker B, Yazel JJ, Short D (1996). Mortality and medical comorbidity among psychiatric patients: a review. Psychiatr Serv.

[2] Cohen BE, Marmar C, Ren L, Bertenthal D, Seal KH (2009). Association of cardiovascular risk factors with mental health diagnoses in Iraq and Afghanistan war veterans using VA health care. JAMA..

[3] Penninx BW (2017). Depression and cardiovascular disease: epidemiological evidence on their linking mechanisms. Neurosci Biobehav Rev.

[4] Roest AM, Martens EJ, de Jonge P, Denollet J (2010). Anxiety and risk of incident coronary heart disease: a meta-analysis. J Am Coll Cardiol.

[5] Krasnova A, Eaton WW, Samuels JF (2019). Antisocial personality and risks of cause-specific mortality: Results from the Epidemiologic Catchment Area study with 27 years of follow-up. Soc psychiatry Psychiatr Epidemiol.

[6] Gjersing L, Bretteville‐Jensen AL (2018). Patterns of substance use and mortality risk in a cohort of ‘hard‐to‐reach’polysubstance users. Addiction..

[7] Whitfield JB, Heath AC, Madden PA, Landers JG, Martin NG (2018). Effects of high alcohol intake, alcohol‐related symptoms and smoking on mortality. Addiction..

[8] Waszczuk MA, Eaton NR, Krueger RF, Shackman AJ, Waldman ID, Zald DH (2019). Redefining phenotypes to advance psychiatric genetics: implications from hierarchical taxonomy of psychopathology. J Abnorm Psychol.

[9] Kim H, Turiano NA, Forbes MK, Kotov R, Kreuger RF, Eaton NR (2021). Internalizing psychopathology and all-cause mortality: a comparison of transdiagnostic vs. diagnosis-based risk prediction. World Psychiatry.

[10] Wolf EJ, Miller MW, Krueger RF, Lyons MJ, Tsuang MT, Koenen KC (2010). Posttraumatic stress disorder and the genetic structure of comorbidity. J Abnorm Psychol.

[11] Miller MW, Fogler JM, Wolf EJ, Kaloupek DG, Keane TM (2008). The internalizing and externalizing structure of psychiatric comorbidity in combat veterans. J Trauma Stress.

[12] Lu AT, Quach A, Wilson JG, Reiner AP, Aviv A, Raj K (2019). DNA methylation GrimAge strongly predicts lifespan and healthspan. Aging.

[13] Horvath S (2013). DNA methylation age of human tissues and cell types. Genome Biol.

[14] Hannum G, Guinney J, Zhao L, Zhang L, Hughes G, Sadda S (2013). Genome-wide methylation profiles reveal quantitative views of human aging rates. Mol Cell.

[15] Wolf EJ, Logue MW, Hayes JP, Sadeh N, Schichman SA, Stone A (2016). Accelerated DNA methylation age: Associations with PTSD and neural integrity. Psychoneuroendocrinology..

[16] McCrory C, Fiorito G, Hernandez B, Polidoro S, O’Halloran AM, Hever A (2021). GrimAge outperforms other epigenetic clocks in the prediction of age-related clinical phenotypes and all-cause mortality. J Gerontology Ser A.

[17] Levine ME, Lu AT, Quach A, Chen BH, Assimes TL, Bandinelli S (2018). An epigenetic biomarker of aging for lifespan and healthspan. Aging.

[18] Hillary RF, Stevenson AJ, Cox SR, McCartney DL, Harris SE, Seeboth A (2021). An epigenetic predictor of death captures multi-modal measures of brain health. Mol Psychiatry.

[19] Katrinli S, Stevens J, Wani AH, Lori A, Kilaru V, van Rooij SJH (2020). Evaluating the impact of trauma and PTSD on epigenetic prediction of lifespan and neural integrity. Neuropsychopharmacology.

[20] Yang R, Wu GW, Verhoeven JE, Gautam A, Reus VI, Kang JI (2021). A DNA methylation clock associated with age-related illnesses and mortality is accelerated in men with combat PTSD. Mol Psychiatry.

[21] Ekaterina P, Yang R, Nier B, Reus V, Hammamieh R, Rampersaud R (2021). “GrimAge,” an epigenetic predictor of mortality, isaccelerated in major depressive disorder. Transl psychiatry.

[22] Han LK, Aghajani M, Clark SL, Chan RF, Hattab MW, Shabalin AA (2018). Epigenetic aging in major depressive disorder. Am J Psychiatry.

[23] Verhoeven JE, Yang R, Wolkowitz OM, Bersani FS, Lindqvist D, Mellon SH (2018). Epigenetic age in male combat-exposed war veterans: associations with posttraumatic stress disorder status. Mol Neuropsychiatry.

[24] Franceschi C, Capri M, Monti D, Giunta S, Olivieri F, Sevini F (2007). Inflammaging and anti-inflammaging: a systemic perspective on aging and longevity emerged from studies in humans. Mech Ageing Dev.

[25] Morrison FG, Logue MW, Guetta R, Maniates H, Stone A, Schichman SA (2019). Investigation of bidirectional longitudinal associations between advanced epigenetic age and peripheral biomarkers of inflammation and metabolic syndrome. Aging.

[26] Levine ME, Lu AT, Bennett DA, Horvath S (2015). Epigenetic age of the pre-frontal cortex is associated with neuritic plaques, amyloid load, and Alzheimer’s disease related cognitive functioning. Aging.

[27] Husain M (2021). Blood tests to screen for Alzheimer’s disease. Brain.

[28] Logue MW, Baldwin C, Guffanti G, Melista E, Wolf EJ, Reardon AF (2013). A genome-wide association study of post-traumatic stress disorder identifies the retinoid-related orphan receptor alpha (RORA) gene as a significant risk locus. Mol Psychiatry.

[29] McGlinchey RE, Milberg WP, Fonda JR, Fortier CB (2017). A methodology for assessing deployment trauma and its consequences in OEF/OIF/OND veterans: the TRACTS longitudinal prospective cohort study. Int J Methods Psychiatr Res.

[30] Blake DD, Weathers FW, Nagy L, Kaloupek D, Klauminzer G, Charney D (1990). A clinical rating scale for assessing current and lifetime PTSD: The CAPS-I. Behav Therapist.

[31] First MB, Spitzer R, Gibbon M, Williams J. Structured clinical interview for axis I DSM-IV disorders--patient edition (SCID-I/P, version 2.0). *Biometrics Research Department, New York State Psychiatric Institute*. 1994.

[32] Spitzer RL, Williams JB, Gibbon M, First MB. *Structured clinical interview for DSM-III-R personality disorders (SCID-II)*. New york State Psychiatric Department; 1989.

[33] Loranger AW. IPDE: International personality disorder examination: DSM-IV and ICD-10 interviews. New York: Cambridge University Press; 1999.

[34] Miller MW, Wolf EJ, Logue MW, Baldwin CT (2013). The retinoid-related orphan receptor alpha (RORA) gene and fear-related psychopathology. J Affect Disord.

[35] Aryee MJ, Jaffe AE, Corrada-Bravo H, Ladd-Acosta C, Feinberg AP, Hansen KD (2014). Minfi: a flexible and comprehensive Bioconductor package for the analysis of Infinium DNA methylation microarrays. Bioinformatics..

[36] Fortin J-P, Triche TJ, Hansen KD (2017). Preprocessing, normalization and integration of the Illumina HumanMethylationEPIC array with minfi. Bioinformatics..

[37] Robbins TW, James M, Owen AM, Sahakian BJ, Lawrence AD, McInnes L (1998). A study of performance on tests from the CANTAB battery sensitive to frontal lobe dysfunction in a large sample of normal volunteers: Implications for theories of executive functioning and cognitive aging. J Int Neuropsychol Soc.

[38] Delis DC, Kaplan E, Kramer JH. Delis-Kaplan executive function system. San Antonio, TX: Pearson. 2001.

[39] Delis DC. California verbal learning test. Adult version manual. San Antonio: Psychological Corporation; 2000.

[40] Wolf EJ, Sadeh N, Leritz EC, Logue MW, Stoop TB, McGlinchey R (2016). Posttraumatic stress disorder as a catalyst for the association between metabolic syndrome and reduced cortical thickness. Biol Psychiatry.

[41] Miller MW, Wolf EJ, Sadeh N, Logue M, Spielberg JM, Hayes JP (2015). A novel locus in the oxidative stress-related gene ALOX12 moderates the association between PTSD and thickness of the prefrontal cortex. Psychoneuroendocrinology..

[42] Lynch FL, Peterson EL, Lu CY, Hu Y, Rossom R, Waitzfelder B (2020). Substance use disorders and risk of suicide in a general US population: A case control study. Addiction Sci Clin Pract.

[43] Krueger RF, Hicks BM, Patrick CJ, Carlson SR, Iacono WG, McGue M (2002). Etiologic connections among substance dependence, antisocial behavior, and personality: modeling the externalizing spectrum. J Abnorm Psychol.

[44] Aquilano K, Baldelli S, Ciriolo MR (2014). Glutathione: new roles in redox signaling for an old antioxidant. Front Pharmacol.

[45] Ndrepepa G, Colleran R, Kastrati A (2018). Gamma-glutamyl transferase and the risk of atherosclerosis and coronary heart disease. Clin Chim Acta.

[46] Whitfield J (2001). Gamma glutamyl transferase. Crit Rev Clin Lab Sci.

[47] Middeldorp J, Hol E (2011). GFAP in health and disease. Prog Neurobiol.

[48] Mayer CA, Brunkhorst R, Niessner M, Pfeilschifter W, Steinmetz H, Foerch C (2013). Blood levels of glial fibrillary acidic protein (GFAP) in patients with neurological diseases. PLoS ONE.

[49] Hol EM, Pekny M (2015). Glial fibrillary acidic protein (GFAP) and the astrocyte intermediate filament system in diseases of the central nervous system. Curr Opin cell Biol.

[50] Pekny M, Pekna M (2016). Reactive gliosis in the pathogenesis of CNS diseases. Biochimic Biophys Acta Mol Basis Dis.

[51] Salat D, Tuch D, Greve D, van der Kouwe AJW, Hevelone ND, Zaleta AK (2005). Age-related alterations in white matter microstructure measured by diffusion tensor imaging. Neurobiol Aging.

[52] Hooker CI, Knight RT. The role of lateral orbitofrontal cortex in the inhibitory control of emotion. In: Zald D, Rauch S, editors. The orbitofrontal cortex. Oxford University Press; New York 2006. p. 307.

[53] Zhang Y, Yu H, Yin Y, Zhou X (2016). Intention modulates the effect of punishment threat in norm enforcement via the lateral orbitofrontal cortex. J Neurosci.

[54] Weiner KS, Zilles K (2016). The anatomical and functional specialization of the fusiform gyrus. Neuropsychologia..

[55] Ding J, Chen K, Chen Y, Fang Y, Yang Q, Lv Y (2016). The left fusiform gyrus is a critical region contributing to the core behavioral profile of semantic dementia. Front Hum Neurosci.

[56] Chen Y, Huang L, Chen K, Ding J, Zhang Y, Yang Q (2020). White matter basis for the hub-and-spoke semantic representation: evidence from semantic dementia. Brain..

[57] Shah M, Kurth F, Luders E (2021). The impact of aging on the subregions of the fusiform gyrus in healthy older adults. J Neurosci Res.

[58] Rubia K, Lim L, Ecker C, Halari R, Giampietro V, Simmons A (2013). Effects of age and gender on neural networks of motor response inhibition: from adolescence to mid-adulthood. Neuroimage..

[59] Vasilenko SA, Evans-Polce RJ, Lanza ST (2017). Age trends in rates of substance use disorders across ages 18–90: Differences by gender and race/ethnicity. Drug Alcohol Depend.

[60] Fitzgerald KN, Hodges R, Hanes D, Stack E, Cheishvili D, Szyf M. Reversal of epigenetic age with diet and lifestyle in a pilot randomized clinical trial. MedRxiv (2020).

