# Correction to: For whom the bell tolls: psychopathological and neurobiological correlates of a DNA methylation index of time-to-death

**DOI:** 10.1038/s41398-023-02340-6

**Published:** 2023-02-15

**Authors:** Sage E. Hawn, Xiang Zhao, Danielle R. Sullivan, Mark Logue, Dana Fein-Schaffer, William Milberg, Regina McGlinchey, Mark W. Miller, Erika J. Wolf

**Affiliations:** 1 National Center for PTSD at VA Boston Healthcare System, Boston, MA USA; 2 Boston University School of Medicine, Department of Psychiatry, Boston, MA USA; 3 Boston University School of Medicine, Department of Medicine, Biomedical Genetics, Boston, MA USA; 4 Boston University School of Public Health, Department of Biostatistics, Boston, MA USA; 5 Translational Research Center for TBI and Stress Disorders (TRACTS) and Geriatric Research, Educational and Clinical Center (GRECC), VA Boston Healthcare System, Boston, MA USA; 6 Department of Psychiatry, Harvard Medical School, Boston, MA USA; 7 Present Address: Department of Psychology, Old Dominion University, Mills Godwin Bldg (134A), Norfolk, VA USA

**Keywords:** Biomarkers, Genetics, Psychology, Neuroscience

Correction to: *Translational Psychiatry* 10.1038/s41398-022-02164-w, published online 24 September 2022

The original version of this article unfortunately contained some mistakes. The authors noticed formatting issues and errors in the tables. These are as follows:

- Table 1: Standard deviation for IL10 (NCPTSD column) should read “.26”, not “26”

**Table 1:** Participant characteristics and descriptive statistics.

	NCPTSD (*N* = 647)		TRACTS (*N* = 434)	
Variable	*M* (SD)	*n* (%)	*M* (SD)	*n* (%)
Demographics				
Sex (male)		407 (62.9)		392 (90.3)
Age	51.85 (10.60)		32.44 (8.63)	
Race				
White		525 (81.1)		322 (74.2)
Black/African American		80 (12.4)		37 (8.5)
Asian		8 (1.2)		6 (1.4)
American Indian/Alaska Native		57 (8.8)		3 (0.7)
Hawaiian/Other Pacific Islander		2 (0.3)		2 (0.5)
Unknown		38 (5.9)		2 (0.5)
Psychiatric				
Lifetime PTSD severity/Dx	60.12 (32.93)	389 (60.1)	69.00 (32.98)	351 (76.8)
# of Trauma types	9.90 (4.26)		1.95 (1.80)	
Lifetime AUD severity/Dx	8.03 (7.81)	376 (58.8)		286 (65.9)
Lifetime Non-AUD SUD severity/Dx	5.24 (9.53)	191 (29.7)		137 (31.6)
Lifetime ASPD severity/Dx	5.19 (5.80)	22 (4.8)		
Neuropsychological				
AGNG				11.56 (9.19)
Stroop^b^				26.59 (12.14)
CVLT-II^c^				48.33 (9.88)
Biological				
CRP (log)			−0.77 (0.29)	
GGT (log)			1.39 (0.24)	
WBC			6.28 (1.71)	
AB40 (log)			2.32 (0.07)	
AB42 (log)			0.91 (0.08)	
BDNF (log)	3.41 (0.38)		3.10 (0.47)	
GFAP (log)			1.79 (0.16)	
NFL (log)			0.72 (0.19)	
NSE (log)	4.32 (0.26)		4.07 (0.21)	
TAU4 (log)			0.16 (0.23)	
PNF (log)			1.41 (0.35)	
Eotaxin (log)			1.61 (0.14)	
IL10 (log)	−0.10 (26)		−0.16 (0.21)	
IL6 (log)	0.19 (0.31)		0.14 (0.27)	
TNF-α (log)	0.53 (0.20)		0.43 (0.13)	

*NCPTSD* National Center for PTSD cohort, *TRACTS* Translational Research Center for TBI and stress disorders cohort, *M* mean, *SD* standard deviation, *PTSD* posttraumatic stress disorder, *dx* diagnosis, *CRP* C-reactive protein, *GGT* gamma-glutamyl transferase, *WBC* total measured white blood cell counts, *AGNG* affective go/no-go task, *CVLT-II* California verbal learning test second edition, *AB40/AB42* amyloid ß 40/42, *BDNF* brain-derived neurotrophic factor, *GFAP* glial fibrillary acidic protein, *NFL* neurofilament light, *NSE* neuron-specific enolase, *PNF* phosphorylated neurofilament heavy, *IL10/6* interleukin 6/10, *TNF-α* tumor necrosis factor.

^a^Raw scores for total commission error = Total positive commissions + total negative commissions.

^b^Scaled scores adjusted for performance = Inhibition – (Color naming + Word reading)/2.

^c^Total words recalled across five learning trials.
- Table 2: NC-PTSD header misaligned

**Table 2:** Psychopathology as a predictor of GrimAge residuals in both cohorts.

*NCPTSD*				
Variable	β	B	*SE*	*p*
Covariates:				
PC1	0.075	9.148	4.616	**0.048**
PC2	−0.023	−2.722	4.443	0.540
PC3	−0.003	−0.397	4.594	0.931
CD8-T	0.064	7.720	4.646	0.097
CD4-T	−0.038	−3.163	3.245	0.330
NK	−0.241	−23.402	3.566	**<0.001**
B-cell	−0.145	−16.815	4.304	**<0.001**
Monocytes	−0.044	−7.601	6.932	0.273
Sex	0.344	3.178	0.359	**<0.001**
Primary Predictors				
Externalizing Factor Score	0.345	0.351	0.044	**<0.001**
Distress Factor Score	−0.120	−0.178	0.095	0.062
Fear Factor Score	0.018	0.008	0.025	0.738
Lifetime PTSD Severity	0.056	0.008	0.006	0.215
***TRACTS***				
**Variable**	**β**	**B**	***SE***	***p***
Covariates:				
PC1	−0.186	−15.297	3.984	**<0.001**
PC2	0.022	2.064	4.485	**<0.001**
PC3	0.053	4.814	4.288	0.262
CD8-T	−0.233	−20.689	4.174	**<0.001**
CD4-T	−0.144	−9.883	3.346	**0.003**
NK	−0.238	−23.583	4.639	**<0.001**
B-cell	−0.051	−7.153	7.010	0.308
Monocytes	−0.108	−14.293	6.473	**0.028**
Sex	−0.150	−1.691	0.527	**0.001**
Primary Predictors:				
Lifetime AUD dx	0.077	0.541	0.339	0.112
Lifetime non-AUD SUD dx	0.104	0.747	0.339	**0.028**
Lifetime PTSD dx	0.149	1.165	1.220	**0.001**

Results based on hierarchical regression models with covariates entered into the first step and psychopathology variables entered in the second. The △*R*^2^ coefficients were significant for the second step across models. *PC* Principal component, *CD* Cluster of differentiation, *NK* Natural killer, *PTSD* Posttraumatic stress disorder, *AUD* Alcohol use disorder, *SUD* Substance use disorder, *dx* Diagnosis, *β* Standardized beta, *B* Unstandardized beta *SE* Standard error for unstandardized beta.
- Table 3: Formatting is misaligned (e.g., there are no results in the right columns)

**Table 3:** GrimAge residuals as a predictor of neuropsychological constructs.

	AGNG			Stroop			CVLT-II		
Variable	β	*p*	*p*_*adj*_	β	*p*	*p*_*adj*_	β	*p*	*p*_*adj*_
Covariates									
Sex	0.011	0.835	–	−0.003	0.955	–	0.033	0.518	–
Age	0.006	0.901	–	−0.041	0.429	–	−0.171	**0.001**	–
Primary Predictor:									
GrimAge Residuals	0.136	0.007	**0.021**	0.052	0.318	.318	−0.122	0.015	**0.023**

Results based on hierarchical regression models with covariates entered into the first step and GrimAge Residuals entered in the second. △*R*^2^ coefficients were significant for the second step across models with significant second step effects. *AGNG* Affective Go/No-Go Task, *CVLT-II* California Verbal Learning Test: Second edition, *PTSD* Posttraumatic stress disorder, *AUD* Alcohol use disorder, *MDD* Major depressive disorder, *p*_*adj*_ *p*-value adjusted for multiple testing by controlling the false discovery rate (FDR) of 5%.
- Covariates is spelled wrong
- Table 3 note refers to entering neuropsychological variables into second step, but it was GrimAge Residuals that were entered into second step
- Table 4: GFAP and NFL results are not in the correct row

**Table 4:** Associations between GrimAge residuals and metabolic, immune, and neurology markers.

	TRACTS			NCPTSD		
Variable	β	*p*	*p*_*adj*_	β	*p*	*p*_*adj*_
**Metabolic, Immune, and Oxidative Stress**						
MetS	0.139	<0.001	**<0.001**	–	–	–
WBC	0.324	<0.001	**<0.001**	–	–	–
GGT	0.161	0.001	**0.003**	–	–	–
**Inflammatory Markers**						
CRP	0.220	<0.001	**<0.001**	–	–	–
Eotaxin	0.108	0.026	0.059	–	–	–
IL10	0.072	0.165	0.264	0.049	0.281	0.281
IL6	0.286	<0.001	**<0.001**	0.341	<0.001	**<0.001**
TNF-alpha	0.077	0.135	0.240	0.156	<0.001	**<0.001**
**Neurologic Markers**						
GFAP	−0.132	0.008	**0.021**	–	–	–
NFL	−0.055	0.266	0.387	–	–	–
pNF	0.001	0.985	0.985	–	–	–
AB42	0.052	0.316	0.421	–	–	–
AB40	0.080	0.122	0.240	–	–	–
NSE	0.043	0.391	0.447	0.072	0.107	0.143
BDNF	0.043	0.386	0.447	0.071	0.114	0.143
Tau4	0.025	0.661	0.705	–	–	–

Results based on hierarchical regression models controlling for age and sex (covariate effects not shown for simplicity). The △*R*^2^ coefficients were significant for the second step across models with significant second step effects. *MetS* Metabolic Syndrome, *WBC* Total measured white blood cell count, *GGT* Gamma-Glutamyl Transferase, *CRP* C-reactive protein, *IL10/IL6* Interleukin 10/6, *TNF-alpha* Tumor necrosis factor, *GFAP* Glial fibrillary acidic protein, *NFL* Neurofilament light, *pNF* Phosphorylated neurofilament heavy chain, *AB42/AB40* Amaloyd beta 40/42, *NSE* Neuron specific enolase, *BDNF* Brain derived neurotrophic factor, *p*_*adj*_ *p*-value adjusted for multiple testing by controlling the false discovery rate (FDR) of.

The correct tables can be found below. The original article has been corrected.

