# Supplementary material for: For whom the bell tolls: psychopathological and neurobiological correlates of a DNA methylation index of time-to-death
Source: Transl Psychiatry. 2022 Sep 24;12:406. doi: 10.1038/s41398-022-02164-w (PMC9509393; doi:10.1038/s41398-022-02164-w)
Supplement: Supplementary file 1 — Supplementary Materials [file 41398_2022_2164_MOESM1_ESM.docx]

Supplementary Materials for “For Whom the Bell Tolls: Psychopathological and Neurobiological Correlates of a DNA Methylation Index of Time-to-Death”

Sage E. Hawn, Xiang Zhao, Danielle R. Sullivan, Mark Logue, Dana Fein-Schaffer, William Milberg, Regina McGlinchey, Mark W. Miller, Erika J. Wolf

*Translational Psychiatry*

**Supplementary Methods**

**DNA Extraction and genotyping**

For both cohorts, DNA was extracted and isolated from peripheral blood samples using a Qiagen AutoPure instrument with Qiagen reagents. Genotypes were assayed on the Illumina HumanOmni2.5-8 BeadChips per manufacturer’s instruction. Chips for both cohorts were counter balanced for sex and PTSD diagnostic status. Concordance between self-report sex and X chromosome homozygosity and cryptic relatedness were checked using PLINK [1]. Ancestry calling was conducted by the pipeline developed by the Psychiatric Genomics Consortium PTSD Workgroup [2] and the ancestry-based principal components were computed from 100,000 randomly chosen common SNPs with MAF>0.05.

**Methylation**

For both cohorts, DNAm data was generated using Illumina Infinium EPIC BeadChips. A panel of SNPs assayed by the EPIC chip were used to check the concordance between DNAm data and genotype data. DNAm data was processed according to a consortium developed pipeline [3]. First, cleaning was conducted using the CpGassoc package [4] and the ChAMP package [5,6] in R. Samples were dropped if they had more than 5% missing data or didn’t meet the probe intensity threshold (> 50% of the experiment-wide mean or with intensity >2,000 arbitrary units). Probes with more than 5% missing data and probes that cross hybridize to sex chromosomes were excluded. Besides, individual probe values were set to missing if not meeting a detection p-value of 0.001. Second, the beta mixture quantile dilation (BMIQ) method implemented in the wateRmelon R package [7] was used to conduct probe normalization. Third, batch effects were removed by an empirical-Bayes batch-correction method (ComBat) using the Bioconductor package sva [8]. Missing data was imputed using a k-nearest neighbor method by the Bioconductor impute package [9]. Further, samples with technical replicates were screened and one of the replicates with the lowest missing rate was retained. Relative proportion of six types of white blood cells (B cells, CD4 cells, CD8 cells, natural killer cells, monocytes and granulocytes) were estimated from the DNAm data using the minfi package [10] in R.

**GrimAge and other DNAm age estimates**

GrimAge was estimated using the EXE file supplied by Lu [11] on the beta values after applying quality control procedures, normalization and imputation. GrimAge calculation requires 30,084 probes with no missing data. First, we extracted the beta values of overlapping probes between our datasets and the required 30,084 probes. Second, the probes that were not present in our data were set to missing and imputed following Horvath’s gold standard approach [11,12]. There were 3,318 missing probes in the discovery cohort and 3,311 and 3,287 missing probes in the baseline and longitudinal TRACTS DNAm data, respectively. Next, we ran the EXE script on our prepared DNAm datasets to generate GrimAge estimates.

Horvath DNAm age was computed on the raw beta values using the R script supplied by Horvath [12]. The script automatically performs normalization and imputation and outputs the DNAm age estimates based on the DNAm at 353 450K probes. 19 probes that were absent on the EPIC array were dropped, which led to 334 CpG sites used for computation. Hannum DNAm age and Levine’s phenotypic age (PhenoAge) were calculated using the post-QC, normalized and imputed DNAm data by computing the product of the beta values from 71 and 513 probes, respectively, times the corresponding effect size estimates [13, 14]. Missing probes were dropped from Hannum age and PhenoAge calculation.

**Psychiatric measures in both cohorts**

In the NCPTSD cohort, all psychiatric diagnostic interviews were videotaped and 30% of them were evaluated for diagnostic reliability. Good to excellent reliability was demonstrated across SCID diagnoses, with a mean kappa of .80 (range: .69-.97) and a mean intraclass correlation coefficient (ICC) of .93 (range: .88-97). PTSD demonstrated excellent reliability (κ=.87, ICC =.97). In TRACTS, diagnostic decisions based on the interviews were reviewed and adjudicated as needed by a panel of expert psychologists.

**Neuropsychological measures in TRACTS**

We examined age-related neurocognitive performance in two domains: inhibitory control and verbal memory. Two measures of inhibitory control were analyzed: the Affective Go/No-go task (AGNG; Robbins et al., 1998) and the color-word interference test (i.e., Stroop) from the Delis–Kaplan Executive Function System (Delis, Kaplan, & Kramer, 2001). AGNG measures response inhibition to negative/positive/neutral words. Total commission errors, which have been widely accepted to index inhibitory control (Schulz et al., 2007) were used as the primary outcome measure. The inhibition subtest of the Stroop task measures inhibition of an automatic response (word reading) in order to generate a less salient incongruent response (color naming). Inhibition scores were adjusted for performance on the word reading and color naming component tests to generate a more homogenous index of inhibition. This index has been previously associated with externalizing phenotypes (Sadeh et al., 2019; Sadeh et al., 2016). Because the Stroop variable was based on standardized (T) scores, greater values indicate greater inhibitory control. Verbal memory recall was assessed using the California Verbal Learning Test: Second Edition (CVLT-II; Delis, 2000), in which participants’ recall of a 16-item word list across five learning trials was summed.

**Metabolic and associated analytes acquired through clinical lab assays in TRACTS**

High-density lipoprotein (HDL) cholesterol, triglycerides, glucose, hemoglobin A1c, and insulin were assayed from fasting overnight blood samples and processed the same day in a clinical laboratory. Two seated blood pressure readings were recorded approximately one minute apart and height, weight, and waist and hip circumference were measured. Following prior work (Wolf, Sadeh, et al., 2016), these variables were included as indicators of latent metabolic variables indexing Blood Pressure, Blood Sugars, and Lipids/Obesity in a confirmatory factor analysis (see Table S1 for fit statistics). These three factors were specified to load on a higher-order latent metabolic pathology factor and subject-level factors scores on each were saved.

One subject was excluded from CRP-related analyses because the obtained CRP value was more than 12 SDs above the sample mean. One outlier on GGT (> 200 and at least 6 *SD*s above the mean) was set to missing on that variable.

**Simoa-derived neurologic and inflammatory analytes in both cohorts**

The Quanterix SP-X and bead-based Simoa® platforms (Quanterix, Billerica, MA) are ultra-sensitive, high-throughput, small molecule digital assays that measure extremely low concentrations of analytes with a high degree of precision using small sample volumes (Rissin et al., 2011). TRACTS blood samples were drawn at the same time as the blood drawn for the aforementioned metabolic markers. Samples were diluted per the manufacturer’s protocol into 96-well plates. Simoa® assays were conducted in batches to avoid multiple freeze-thaw cycles. Assays were run in duplicate. Samples were excluded (3.5% - 26.1%, varying by marker) for the following reasons: coefficient of variation (CV) > 20% or duplicate not available. Samples with a concentration<lower limit of quantification (LLOQ) were set to the LLOQ. After exclusion criteria were applied, the mean of the duplicate runs was computed and used in analyses.

In NCPTSD, Simoa® analytes were obtained via the Quanterix Accelerator Lab (Quanterix Corporation, Billerica, MA) from frozen plasma aliquots that had been stored at -80C from the date the samples were obtained. Samples were thawed and diluted per manufacturer’s specifications and then underwent centrifugation to remove particulates and debris. Calibration was conducted with reference samples. Samples were pipetted into 96 well plates. All neuropathology markers were tested in duplicate and 20% of the inflammatory molecules were also tested in duplicate. Inflammatory molecules were obtained via the multiplex CorPlex Human Cytokine Panel 1 on the SP-X Imaging and Analysis System and neuropathology markers with the HD-1 Analyzer. Quality control procedures included evaluation of average enzyme per bead and CV. Samples that did not pass quality control procedures were rerun where possible, with the goal of minimizing freeze/thaw cycles. Samples were excluded (0% - 4.5%, varying by marker) for the following reasons: coefficient of variation > 25% or duplicate not available (neuropathology markers only). Samples with a concentration < LLOQ were set to the LLOQ. Samples with a concentration > upper limit of quantification (ULOQ) were set to the ULOQ.

**MRI data acquisition and processing (TRACTS cohort)**

Structural imaging data were available for 389 participants in the TRACTS cohort. For the first 361 participants recruited into the study, two Magnetization Prepared Rapid Gradient Echo (MP-RAGE) T1-weighted structural scans were acquired on a 3-Tesla Siemens Trio whole-body TIM Trio MRI scanner using the following parameters: TR=2530ms, TE=3.32ms, flip angle=7°, FOV=256, Matrix=256x256, voxel size=1mm^3^. After a scanner upgrade, the remaining 28 participants had two MP-RAGE T1-weighted structural scans acquired on a Siemens Prisma scanner with Syngo D13D software with the following parameters: TR=2530ms, TE=3.35ms, flip angle=7°, FOV=256, Matrix=256x256, voxel size=1mm^3^. A scanner flag was included in analyses as a covariate to account for potential scanner differences. The two T1-weighted structural scans were averaged to create a single high contrast-to-noise image. A second MP-RAGE was unavailable for seven individuals. For those individuals, cortical thickness analyses were completed with a single MP-RAGE.

Cortical thickness analysis was performed using the FreeSurfer image analysis suite (version 5.3, http://surfer.nmr.mgh.harvard.edu). The cortical surface was reconstructed and volumetric segmentation was conducted on the images (Dale, Fischl, & Sereno, 1999; Fischl & Dale, 2000; Fischl, Sereno, Tootell, & Dale, 1999). To extract the mean cortical thickness for regions of interest, cortical parcellations were created for each individual via FreeSurfer (2002) with the Desikan-Killiany parcellation (34 regions per hemisphere; Desikan et al., 2006) and the mean cortical thickness for specific parcellations (i.e., lateral orbitofrontal cortex and posterior cingulate cortex) was extracted for each individual. Parcellations were manually checked and edited for accuracy by members of the research team.

**Supplementary Results**

**Sensitivity Analyses**

We tested for a variety of potential confounds, depending on the outcome variable of interest. Not all variables were available in both datasets so covariate sensitivity analyses differ somewhat by cohort.

***NCPTSD Cohort: GrimAge residuals, PTSD, and psychopathology***

The associations between the broad externalizing factor (b=.35, std β=.35, *p*<.001) and non-AUD SUD (b = .11, std β = .21, *p* < .001) with GrimAge residuals remained significant after additionally controlling for income. TBIs were not assessed in the NCPTSD cohort.

***TRACTS Cohort: GrimAge residuals and externalizing disorders***

The non-alcohol use SUD (b = .67, std β = .09, *p* = .048) and lifetime PTSD diagnosis (b = 1.17, std β = .15, *p* = .001) effects remained significant when included in the same model, along with number of lifetime TBIs. Income was not available in the TRACTS cohort.

***TRACTS Cohort: GrimAge residuals and neuropsychological constructs***

The effect of GrimAge residuals on the AGNG task remained significant (b=.32, std β=.11, *p*=.026) when additionally controlling for the effects of lifetime PTSD, individual externalizing diagnoses, and number of lifetime TBIs and in a sensitivity analysis using GrimAge residualized for age and estimated WBC composition (b=.37, std β=.13, *p*=.012).

The effect of GrimAge residuals on CVLT just missed the threshold for significance (b=-.29, std β=-.09, *p*=.065) when additionally controlling for the effects of lifetime PTSD, AUD, non-AUD SUD, and number of lifetime TBIs, though survived a sensitivity analysis using GrimAge residualized for age and estimated WBC composition (b=-.36, std β=-.11, *p*=.027).

***TRACTS Cohort: GrimAge residuals and metabolic, immune, and neurology analytes***

The effect of GrimAge residuals on the MetS factor scores (b = .002, β = .123, *p* < .001), CRP (b = .019, β = .209, *p* < .001), GGT (b = .010, β = .140, *p =* .007), total measured WBCs (b = .157, β = .304, *p <* .001), GFAP (b = -.005, β = -.108, *p =* .032), and IL-6 (b = .021, β = .260, *p <* .001) remained significant when additionally controlling for the effects of lifetime PTSD, individual externalizing diagnoses, and number of lifetime TBIs and in sensitivity analyses using GrimAge residualized for age and estimated WBC composition: MetS factor scores (b = .001, β = .105, *p* = .002), CRP (b = .014, β = .150, *p* = .002), GGT (b = .011, β = .140, *p =* .006), total measured WBCs (b = .102, β = .188, *p <* .001), GFAP (b = -.006, β = -.117, *p =* .020), and IL-6 (b = .018, β = .210, *p <* .001).

***NCPTSD Cohort: GrimAge residuals and immune and neurology markers***

The effect of GrimAge residuals on IL-6 (b = .024, β = .344, *p <* .001) and TNF-α (b = .008, β = .184, *p =* .002) remained significant when additionally controlling for the effects of lifetime PTSD and individual externalizing severity, and in sensitivity analyses using GrimAge residualized for age and estimated WBC composition: IL-6 (b = .019, β = .272, *p <* .001) and TNF-α (b = .008, β = .165, *p <* .001).

***TRACTS Cohort: GrimAge residuals and neural integrity***

The negative association between GrimAge residuals and cortical thickness in the right lateral orbitofrontal cortex remained significant in the follow-up sensitivity analysis, which featured GrimAge residualized on age and estimated cell type composition (b=-0.005, *p*=.021) and when additionally controlling for the effects of lifetime PTSD, AUD, non-AUD SUD, and number of lifetime TBIs (b=-.006, *p=*.008).

The negative association between GrimAge residuals and whole brain cortical thickness in the left fusiform gyrus also remained significant in a follow-up sensitivity analysis which featured the extracted significant cortical thickness region and GrimAge residualized on age and estimated cell type composition (b=-.014, *p*<.001) and when controlling for the effects of lifetime PTSD, AUD, non-AUD SUD, and lifetime number of TBIs (b=-.016, *p<*.001).

**Supplementary Tables**

Table S1.

*Fit statistics for psychopathology factors in the discovery cohort and MetS factors in the TRACTS cohort*

| Model | χ^2^ (df) | RMSEA | SRMR | CFI | TLI |
| --- | --- | --- | --- | --- | --- |
| MetS Measurement Model | 149.44 (31)^*^ | 0.103 | 0.058 | 0.95 | 0.93 |
| 3-Factor Psychopathology CFA | 53.11 (40) | 0.03 | 0.03 | 0.99 | 0.98 |

*Note.* MetS = metabolic syndrome; CFA = confirmatory factor analysis; RMSEA = root mean square error of approximation; SRMR = standardized root mean square residual; TLI = Tucker-Lewis Index; ^*^ *p* < .001.

Table S2.

*Descriptive statistics for variables used to create factor latent psychopathology scores in the discovery cohort*

| **Variable** | ***M* (*SD*)** |
| --- | --- |
| **EXTERNALIZING** |  |
| Lifetime ASPD Severity | 5.19 (5.80) |
| Lifetime AUD Severity | 8.03 (7.81) |
| Lifetime Non-AUD SUD Severity | 5.24 (9.53) |
| **DISTRESS** |  |
| Lifetime Depression Severity | 9.99 (5.85) |
| Lifetime GAD Severity | 5.86 (5.26) |
| Lifetime Dysthymia Severity | 5.07 (4.66) |
| **FEAR** |  |
| Lifetime Panic Disorder Severity | 11.05 (9.92) |
| Lifetime Agoraphobia Severity | 8.25 (10.08) |
| Lifetime OCD Severity | 1.07 (2.78) |
| Lifetime Specific Phobia Severity | 3.22 (3.57) |
| Lifetime Social Phobia Severity | 1.95 (3.28) |

*Note.* PTSD = Posttraumatic stress disorder; OCD = obsessive-compulsive disorder; GAD = generalized anxiety disorder; AUD = alcohol use disorder; ASPD = antisocial personality disorder; Mean and standard deviation calculated using only the participants used in analyses.

Table S3.

*Correlations Among DNAm Age Estimates and Chronological Age*

| Variable | 1 | 2 | 3 | 4 | 5 | 6 | 7 | 8 | 9 |
| --- | --- | --- | --- | --- | --- | --- | --- | --- | --- |
| 1. Chronological Age | 1.00 | .890^**^ | .870^**^ | .854^**^ | .804^**^ | .000 | .000 | .000 | .000 |
| 2. Grim Age | .884^**^ | 1.00 | .830^**^ | .775^**^ | .826^**^ | .456^**^ | .030 | .113^*^ | .185^**^ |
| 3. Hannum Age | .879^**^ | .831^**^ | 1.00 | .840^**^ | .902^**^ | .122^*^ | .186^**^ | .493^**^ | .341^**^ |
| 4. Horvath Age | .902^**^ | .825^**^ | .872^**^ | 1.00 | .799^**^ | .034 | .521^**^ | .197^**^ | .190^**^ |
| 5. Levine Age | .848^**^ | .827^**^ | .841^**^ | .871^**^ | 1.00 | .241^**^ | .217^**^ | .411^**^ | .595^**^ |
| 6. Grim Age Residuals  7. Horvath Age Residuals | .000  .002 | .468^**^  .066 | .115^**^  .185^**^ | .060  .433^**^ | .165^**^  .248^**^ | 1.00  .138^**^ | .066  1.00 | .248^**^  .378^**^ | .406^**^  .364^**^ |
| 8. Hannum Age Residuals | -.002 | .112^**^ | .475^**^ | .164^**^ | .200^**^ | .242^**^ | .384^**^ | 1.00 | .691^**^ |
| 9. Levine Age Residuals | -.001 | .145^**^ | .180^**^ | .199^**^ | .529^**^ | .312^**^ | .464^**^ | .380^**^ | 1.00 |

*Note.* NCPTSD sample values are below the diagonal; TRACTS sample values are above the diagonal; ^**^ *p* < .01, ^*^ *p* < .05

**Supplementary References**

1. Purcell, S., Neale, B., Todd-Brown, K., Thomas, L., Ferreira, M. A., Bender, D., et al. (2007). PLINK: a tool set for whole-genome association and population-based linkage analyses. *The American journal of human genetics*, 81(3), 559-575.
2. Logue, M. W., Amstadter, A. B., Baker, D. G., Duncan, L., Koenen, K. C., Liberzon, I., et al. (2015). The Psychiatric Genomics Consortium Posttraumatic Stress Disorder Workgroup: posttraumatic stress disorder enters the age of large-scale genomic collaboration. *Neuropsychopharmacology*, 40(10), 2287-2297.
3. Ratanatharathorn, A., Boks, M. P., Maihofer, A. X., Aiello, A. E., Amstadter, A. B., Ashley‐Koch, A. E., et al. (2017). Epigenome‐wide association of PTSD from heterogeneous cohorts with a common multi‐site analysis pipeline. *American Journal of Medical Genetics Part B: Neuropsychiatric Genetics*, 174(6), 619-630.
4. Barfield, R. T., Kilaru, V., Smith, A. K., & Conneely, K. N. (2012). CpGassoc: an R function for analysis of DNA methylation microarray data. *Bioinformatics*, 28(9), 1280-1281.
5. Tian, Y., Morris, T. J., Webster, A. P., Yang, Z., Beck, S., Feber, A., et al. (2017). ChAMP: updated methylation analysis pipeline for Illumina BeadChips. *Bioinformatics*, 33(24), 3982-3984.
6. Morris, T. J., Butcher, L. M., Feber, A., Teschendorff, A. E., Chakravarthy, A. R., Wojdacz, T. K., et al. (2014). ChAMP: 450k chip analysis methylation pipeline. *Bioinformatics*, 30(3), 428-430.
7. Pidsley, R., Wong, C. C., Volta, M., Lunnon, K., Mill, J., & Schalkwyk, L. C. (2013). A data-driven approach to preprocessing Illumina 450K methylation array data. *BMC genomics*, 14(1), 1-10.
8. Leek, J. T., Johnson, W. E., Parker, H. S., Jaffe, A. E., & Storey, J. D. (2014). *sva: Surrogate Variable Analysis*. R package version 3.10. 0. DOI, 10, B9.
9. Hastie, T., Tibshirani, R., Narasimhan, B., & Chu, G. (2020). *impute: Imputation for microarray data.* R package version 1.64.0.
10. Aryee, M. J., Jaffe, A. E., Corrada-Bravo, H., Ladd-Acosta, C., Feinberg, A. P., Hansen, K. D., et al. (2014). Minfi: a flexible and comprehensive Bioconductor package for the analysis of Infinium DNA methylation microarrays. *Bioinformatics*, 30(10), 1363-1369.
11. Lu, A. T., Quach, A., Wilson, J. G., Reiner, A. P., Aviv, A., Raj, K., et al. (2019). DNA methylation GrimAge strongly predicts lifespan and healthspan. *Aging (Albany NY)*, 11(2), 303.
12. Horvath, S. (2013). DNA methylation age of human tissues and cell types. *Genome biology*, 14(10), 1-20.
13. Hannum, G., Guinney, J., Zhao, L., Zhang, L., Hughes, G., Sadda, S., et al.. (2013). Genome-wide methylation profiles reveal quantitative views of human aging rates. *Molecular cell*, 49(2), 359-367.
14. Levine, M. E., Lu, A. T., Quach, A., Chen, B. H., Assimes, T. L., Bandinelli, S., et al. (2018). An epigenetic biomarker of aging for lifespan and healthspan. *Aging (Albany NY),* 10(4), 573.
